# Mesonephric-like Adenocarcinoma of the Ovary: Clinicopathological and Molecular Characteristics

**DOI:** 10.3390/diagnostics12020326

**Published:** 2022-01-27

**Authors:** Hyun Hee Koh, Eunhyang Park, Hyun-Soo Kim

**Affiliations:** 1 Department of Pathology and Translational Genomics, Samsung Medical Center, Sungkyunkwan University School of Medicine, Seoul 06351, Korea; hyunhhee0301.koh@samsung.com; 2 Department of Pathology, Severance Hospital, Yonsei University College of Medicine, Seoul 03722, Korea

**Keywords:** ovary, mesonephric-like adenocarcinoma, immunohistochemistry, targeted sequencing

## Abstract

Mesonephric-like adenocarcinoma (MLA) arising in the ovary is a rare malignant tumor of the female genital tract. Although the clinicopathological and molecular characteristics of uterine MLA have been accumulated, those of ovarian MLA have not been firmly clarified. In this study, we investigated the clinicopathological, immunohistochemical, and genetic features of five ovarian MLAs. A review of electronic medical records and pathology slides, immunostaining, and targeted sequencing was performed. On imaging, ovarian MLA presented as either a mixed solid and cystic mass or a purely solid mass. One, three, and one patient were diagnosed as having FIGO stage IA, IC, and II MLA, respectively. Four patients with stage IC–II tumor underwent post-operative adjuvant chemotherapy. Three of the four patients whose follow-up information was available did not experience recurrence. In contrast, the remaining patient with stage IA tumor who did not receive any adjuvant treatment developed multiple metastatic recurrences at post-operative 13 months. Histologically, ovarian MLAs characteristically displayed architectural diversity, compactly aggregated small tubules, and eosinophilic intraluminal secretions. Four tumors were found to be associated with endometriotic cysts. Two cases showed some areas of high-grade nuclear atypia, brisk mitotic activity, and necrosis. Immunohistochemically, all cases showed positive immunoreactivities for at least three of the four examined mesonephric markers (GATA3, PAX2, TTF1, and CD10), lack of WT1 expression, non-diffuse p16 immunoreactivity, and wild-type p53 immunostaining pattern. Targeted sequencing analysis revealed that all four examined cases harbored pathogenic *KRAS* mutations: p.G12V (2/4); p.G12D (1/4); and p.G12C (1/4). In addition, we reviewed the previous literature reporting 60 cases of ovarian MLA. Our findings corroborate those of the previous data regarding the clinical presentation, histological features, immunophenotypes, and molecular alterations. Our observations should encourage pathologists to recognize and accurately diagnose this rare but distinct entity.

## 1. Introduction

Mesonephric tubules and ducts are precursors of the male genital tract present during human embryogenesis [1]. In men, it gives rise to the internal male genitalia, including the epididymis, vas deferens, seminal vesicle, and efferent ductule of the testis; whereas in women, it regresses with some remnants persisting in the broad ligament and the lateral wall of uterine cervix and vagina [2]. Mesonephric adenocarcinoma (MA) is a rare malignant tumor of the female genital tract that comprises less than 1% of all gynecological malignancies [3]. It is thought to arise from the embryonal remnants of mesonephric tubules and ducts, and is typically located in the uterine cervix and vagina; however, several cases of malignant mesonephric lesions arising in the uterine corpus and adnexa have also been reported [4]. Within this context, MA of the upper female genital tract has been referred to as mesonephric-like adenocarcinoma (MLA) because the association with mesonephric remnants has not been firmly established [3,4].

We recently experienced several cases of MLA arising in the ovary. Although some case series and individual case reports of ovarian MLA have been published in the literature [4,5,6,7,8,9,10,11,12,13,14], the clinicopathological and molecular characteristics have not yet been clarified. In this study, we investigated the clinical presentation, histological features, immunophenotype, and genetic characteristics of ovarian MLAs. Our observations will allow pathologists to recognize and accurately diagnose this rare entity.

## 2. Materials and Methods

### 2.1. Case Selection and Clinicopathological Data Collection

We found five cases from surgical pathology archives, using the combination of keywords ‘mesonephric’, ‘mesonephric-like’, ‘carcinoma’, ‘adenocarcinoma’, ‘adnexa’, ‘ovary’, and ‘meso-ovarium’. Clinical information, including age of patient at diagnosis, previous gynecological history, presenting symptom, imaging finding, serum levels of cancer antigen (CA) 125 and CA 19-9, pre-operative clinical impression, surgical procedure, post-operative treatment, recurrence, metastasis, current status, and survival period, was obtained from the electronic medical records and pathology reports. Two board-certified gynecological pathologists thoroughly reviewed all available hematoxylin and eosin-stained slides using light microscopy. Pathological information, including the location and greatest dimension of tumor, ovarian surface extension, lymphovascular space invasion (LVSI), salpingeal and uterine extension, pelvic and extrapelvic peritoneal metastases, International Federation of Gynecology and Obstetrics (FIGO) stage, histological growth patterns, severe nuclear pleomorphism, mitotic count per 10 high-power fields (HPFs), and coagulative tumor cell necrosis, were collected. The most representative slide was selected to perform immunostaining and targeted sequencing.

### 2.2. Immunohistochemical Staining

Four-micrometer-thick, formalin-fixed, paraffin-embedded (FFPE) slices were deparaffinized and rehydrated using a xylene and alcohol solution. Immunostaining was performed using automated instruments [15,16,17,18,19,20,21,22,23,24,25,26]. After antigen retrieval, the slices were incubated with the primary antibodies listed in Table 1. After chromogenic visualization, the slices were counterstained with hematoxylin. Appropriate controls were concurrently stained to validate the staining method. Normal salpingeal tissue (for PAX8 and WT1), invasive breast carcinoma of no specific type (for GATA3), normal thyroid tissue (for TTF1), normal proliferative endometrium (for PAX2, CD10, ER, PR, PTEN, MLH1, PMS2, MSH2, and MSH6), and ovarian high-grade serous carcinoma (HGSC; for p53 and p16) were used as positive controls. Negative controls were prepared by substituting non-immune serum for primary antibodies, resulting in no detectable staining.

### 2.3. Immunohistochemical Interpretation

For PAX8, PAX2, GATA3, WT1, ER, PR, and TTF1, staining in the nuclei was interpreted as positive expression [15]. The intensity of positive staining was graded as strong, moderate, and weak. The proportion of staining was established as diffuse when 50% or more of the tumor cells were stained, and focal when less than 50% were stained. For PTEN, at least weak staining in the cytoplasm was interpreted as preserved expression; whereas loss of expression was defined as the complete absence of cytoplasmic PTEN immunoreactivity. For CD10, we examined the subcellular localization of positive immunoreactivity. The p53 immunostaining pattern was interpreted as a mutation pattern when one of the following staining patterns was observed: diffuse and strong nuclear immunoreactivity in ≥75% of the tumor cells (overexpression pattern); no nuclear immunoreactivity in any of the tumor cells (complete absence pattern); and unequivocal cytoplasmic staining, which is accompanied by a variable nuclear staining (cytoplasmic pattern) [27]. In contrast, p53 expression was interpreted as a wild-type pattern if a variable proportion of nuclear expression with mild-to-moderate staining intensity was present [28]. The p16 immunostaining pattern was interpreted as diffuse and strong when p16 expression was continuous and strong, nuclear, or nuclear plus cytoplasmic staining. All other p16 immunostaining patterns, described as focal nuclear or cytoplasmic staining, were interpreted as patchy [15,29,30,31]. The expression of four mismatch repair (MMR) proteins were evaluated in three categories as retained, loss, and subclonal loss [32,33].

### 2.4. DNA Extraction, Library Preparation, and Targeted Sequencing

DNA was isolated from 10-μm thick slices of FFPE tissue using a sterile 26-gauge needle and RecoverAll Multi-Sample RNA/DNA Isolation Workflow (Thermo Fisher Scientific, Waltham, MA, USA). The tumor tissue was obtained by manual microdissection and subjected to DNA extraction for library preparation. The normal tissues of each case were obtained from the adjacent non-neoplastic area. DNA was quantified using the Qubit 2.0 Fluorometer (Thermo Fisher Scientific). DNA libraries were prepared as previously described [34,35]. They were generated from 20 ng of DNA per sample using an Ion AmpliSeq Library Kit 2.0 (Thermo Fisher Scientific) and Oncomine Comprehensive Assay (OCA) v1 panel (Thermo Fisher Scientific). Libraries were quantified using the Ion Library Universal Quantification Kit (Thermo Fisher Scientific). The OCA v1 panel (Thermo Fisher Scientific) included 143 genes, of which 73 oncogenes were interrogated for mutational hotspots and 26 tumor-suppressor genes were interrogated for all exons. Consecutively, a 60 pmol/l pool of DNA library was used to prepare the templated Ion Sphere Particle (Thermo Fisher Scientific). Sequencing was performed using the Ion 540 Kit-Chef (Thermo Fisher Scientific) and Ion S5 system (Thermo Fisher Scientific). Sequencing data of approximately 200 bp reads were generated after 500 flow runs.

### 2.5. Bioinformatics and Data Analysis Pipeline

Analysis of the sequencing data was performed using Torrent Suite Software v5.2.2 (Thermo Fisher Scientific). This workflow was created by adding a custom hotspots Browser Extensible Data file to report mutations of interest, and a custom CNV baseline using the manufacturer’s default workflow as described previously [34,35]. The pipeline included signaling processing, base calling, quality score assignment, adapter trimming, read mapping to the human genome assembly GRCh37, quality control of mapping, coverage analysis with downsampling, and variant calling. The identification of variants was performed using the Torrent Variant Caller plug-in and Ion Reporter Software v5.2 (Thermo Fisher Scientific), and coverage maps were generated using the Coverage Analysis plug-in (Thermo Fisher Scientific). Additionally, ANNOVAR was used for functional annotation of identified single nucleotide polymorphisms (SNPs) to investigate their genomic locations and variation [36]. To eliminate error artifacts, sequence data were visually confirmed using the Integrative Genomics Viewer (Broad Institute, Cambridge, MA, USA). This workflow was able to report SNPs and indels in as low as 1% of the variant allele fraction. Based on the results of a feasibility study, the variant allele fraction threshold was established at 5%.

## 3. Results

### 3.1. Clinical Characteristics

**Figure 1:**
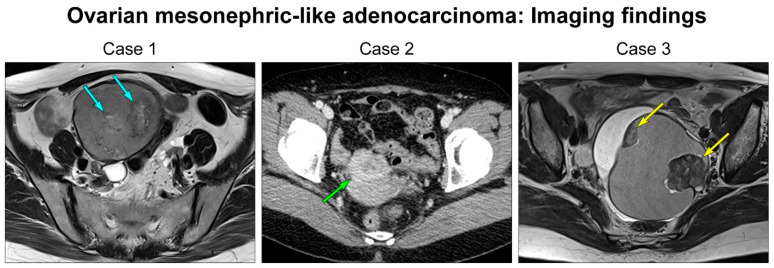
Imaging and gross findings of ovarian MLA. Pelvic magnetic resonance imaging (cases 1 and 3) and computed tomography (case 2) revealed cystic ovarian masses containing internal solid nodules (blue, green, and yellow arrows).

The five patients ranged in age from 42–61 years (mean, 53 years) (Table 2). One (case 1) and two (cases 2 and 3) patients had uterine adenomyosis and leiomyomata, respectively. One patient (case 3) underwent total hysterectomy for uterine leiomyomata. Four patients presented with pelvic mass (cases 1, 4, and 5) or abdominal distension (case 3). Imaging findings were available in all patients. Four (cases 1–4) patients had mixed solid and cystic ovarian masses, described as ovarian cystic tumors possessing internal solid nodules, small enhancing nodules, or solid components. In contrast, one tumor (case 5) appeared as a purely solid mass that involved the lateral uterine wall and adnexa. The greatest dimension of tumor ranged from 4.8–10.9 cm (mean, 7.2 cm) on imaging. Figure 1 depicts representative magnetic resonance and computed tomography images of ovarian MLA. Serum levels of CA 125 and CA 19-9 were examined in four and three patients, respectively. Serum CA 125 was elevated up to 72.5 U/mL (case 1) and 108.8 U/mL (case 5) in two patients, respectively, and one patient (case 1) showed an elevated serum CA 19-9 (80.1 U/mL). Preoperative clinical impressions included ovarian borderline tumor or carcinoma (cases 2 and 3), ovarian carcinoma (cases 1 and 4), and uterine leiomyoma or leiomyosarcoma (case 5). All patients underwent primary debulking surgery. None of the patients underwent pre-operative neoadjuvant chemotherapy. Initial pathological FIGO stages were IA (1/5), IC (3/5), and IIB (1/5). All except one patient with stage IA disease underwent post-operative adjuvant chemotherapy with six cycles of taxane and platinum-based combination regimen.

Follow-up information was available for all except one patient (case 4), who was lost to follow-up during the first cycle of post-operative chemotherapy. Three patients who underwent post-operative chemotherapy did not develop local or metastatic recurrence, with a mean disease-free survival time of 31 months (range, 11–53 months). At the end of the follow-up, these patients were alive without evidence of disease. In contrast, the remaining one patient (case 1), who was initially diagnosed as stage IA MLA and did not receive any post-operative treatment, developed abdominopelvic peritoneal recurrence and distant metastases to the liver and bilateral lungs at 13 months post-operatively. Regardless, the patient underwent secondary debulking surgery and post-operative chemotherapy, the disease progressed during treatment, and she died 39 months after the initial surgery.

### 3.2. Pathological Characteristics

Five MLAs arose in the right (2/5) or left (3/5) ovary (Table 3). Mean greatest dimension was 7.0 cm (range, 4.7–11.0 cm). Two (cases 2 and 3) tumors involved the ovarian surface, and two patients (cases 2 and 4) harbored malignant cells in the intraoperative peritoneal washing. One tumor (case 5) showed uterine involvement, while none of the cases showed salpingeal involvement or LVSI. One patient (case 5) had pelvic peritoneal metastases while there was no extra-pelvic peritoneal metastasis at the time of initial diagnosis. Histologically, the tumor exhibited diverse architectural patterns, including compact aggregation or fusion of small tubules, inter-anastomosing cords and solid sheets of spindle-shaped cells, endometrioid-like glands and ducts, papillary and micropapillary architecture, cribriform structure, comedonecrosis-like pattern, sex cord-like pattern, and randomly scattered small, angulated glands associated with prominent desmoplastic stroma. Of these, the two most predominant patterns were tubular (3/5) and ductal (2/5). Four cases displayed either endometriotic cyst (cases 2–4) or endometriosis (cases 5) located adjacent to the tumor tissue.

**Figure 2:**
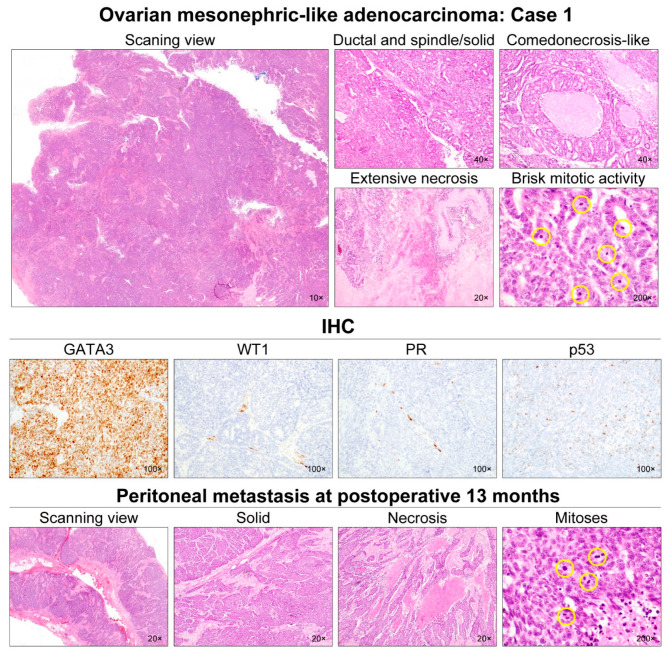
Histological features of ovarian MLA: Case 1. The ovarian mass was a 7.5-cm cystic lesion with some internal mural nodules. At scanning magnification, the mural nodules appeared solid and deeply basophilic due to hypercellularity. They consisted of adenocarcinoma, showing ductal and solid growth patterns. Most of the tumor cells formed a complex cribriform and solid architecture with slit-like glandular spaces. We noted some foci of intraluminal necrosis and karyorrhexis within the cribriform architecture, resembling comedonecrosis observed in ductal carcinoma in situ of the breast. Even though most of the tumor cells exhibited mild-to-moderate nuclear pleomorphism, some areas showed severe nuclear pleomorphism, extensive coagulative tumor cell necrosis, and brisk mitotic activity (27/10 high-power fields). Immunostaining revealed that the tumor cells were completely negative for WT1, ER, PR, and p16; p53 expression pattern was wild-type. Since the tumor was confined within the right ovary (FIGO stage IA), the patient received no further treatment. However, at 13 months post-operatively, she developed multiple metastases in the abdominopelvic peritoneum, liver, and lungs. The metastatic lesions displayed solid growth pattern with scattered foci of coagulative tumor necrosis. Similar to the primary tumor, the metastatic tumor cells showed severe nuclear pleomorphism. Mitotic count (25/10 high-power fields) was similar to that of the primary tumor. The yellow circles are intended to highlight the mitotic figures, which just appear very small dark spots.

**Figure 3:**
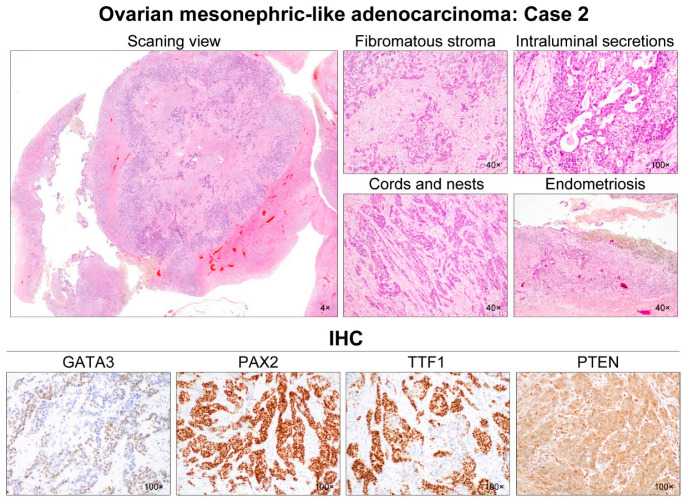
Histological features of ovarian MLA: Case 2. A 4.7-cm ovarian mass contained small, nodular components confined to the cyst. The mural nodules consisted of solid tumor tissue showing infiltrating small tubules in the background of the fibromatous stroma. These tubules were haphazardly arranged and anastomosed with each other, forming a cribriform or trabecular architecture. In addition to the small tubular pattern, the tumor cells formed irregular-shaped nests and cords, separated by thin intervening fibrous stroma. Hyaline-like eosinophilic secretions were occasionally noted within the tubular lumina and dilated duct-like structures. No severe nuclear pleomorphism was identified. The cystic lining was primarily composed of endometrial-like glands and stroma, the latter of which was occupied by hemosiderin-laden macrophages. Immunohistochemically, the tumor cells were focally positive for GATA3 with moderate staining intensity. PAX2 immunoreactivity was uniform and intense throughout the tumor. TTF1 expression was also diffuse and strong in most of the tumor cells. Hormone receptor expressions were absent, and loss of PTEN expression was not observed.

**Figure 4:**
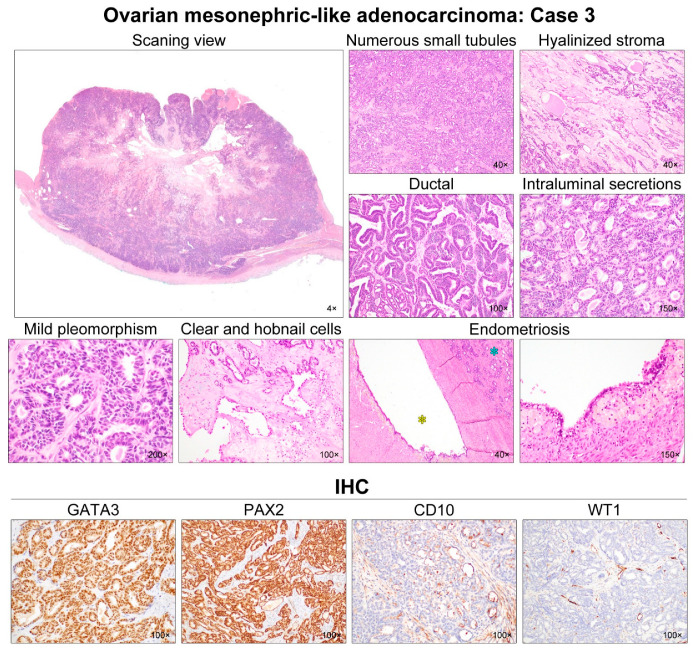
Histological features of ovarian MLA: Case 3. Scanning magnification revealed that the solid component was a hypercellular tumor, with areas of cystic change and degeneration in the central portion. Numerous small tubules were compactly aggregated and merged. The amount of intervening stroma was small, but the central portion had some microscopic areas of stromal degeneration and hyaline fibrosis. An endometrioid-like glandular structure (i.e., ductal growth pattern), accompanied with focal maze-like architecture, comprised approximately one-third of the entire tumor volume. These tubules and endometrioid-like glands were lined by cuboidal and columnar epithelial cells, respectively, and possessed pale or dense eosinophilic intraluminal secretions. Most tumor cell nuclei demonstrated intermediate-grade atypia characterized by mild-to-moderate pleomorphism and hyperchromasia, with rare mitotic figures. In a few foci, dilated glands were lined by a single layer of tumor cells with clear-to-eosinophilic cytoplasm and a hobnail appearance. Their nuclei were similar in size and shape to those of the adjacent tubules. The intervening stroma showed sparse inflammatory infiltrate, and focal myxoid and hyaline degeneration. An endometriotic cyst (yellow asterisk) was observed adjacent to the tumor tissue (blue asterisk). The lining epithelium of the endometriotic cyst underwent extensive tubal metaplasia. The endometrial stroma was filled with hemosiderin-laden macrophages. Immunostaining revealed that the tumor cells were diffusely and strongly positive for GATA3 and PAX2. CD10 expression, along the luminal surface of the tubules and ducts, was characteristic of MLA. Lack of WT1 expression excluded the possibility of serous carcinoma.

**Figure 5:**
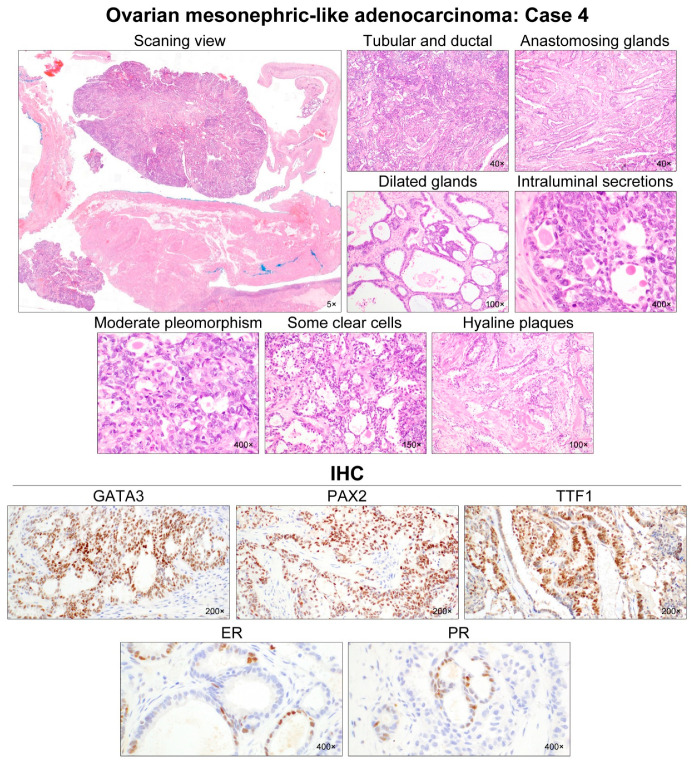
Histological features of ovarian MLA: Case 4. The ovarian mass was solid and cystic, with mural nodules protruding into the cystic lumen. The cystic wall consisted of fibromuscular connective tissue, whereas the solid mural nodules were composed of adenocarcinoma. Two predominant growth patterns were tubular and ductal. Small tubules were compactly aggregated and admixed with inter-anastomosing glands. Cystically dilated glands were often seen. Hyaline-like intraluminal substances were frequently identified in the tubules and glands. The tumor cell nuclei exhibited mild-to-moderate pleomorphism. In addition, angulated ductal structures traversed by thick bands of hyaline collagen were observed. In a few foci, some tubules lined by a few layers of cuboidal or hobnail cells with clear cytoplasm raised the possibility of clear cell carcinoma (CCC). However, severe nuclear pleomorphism, prominent nucleoli, and myxoid stromal cores, characteristic of CCC, were absent. The tumor cells were strongly positive for GATA3, PAX2, and TTF1. We noted that some tumor cells were positive for ER and PR, with moderate staining intensity. Although hormone receptor immunoreactivities were not typical of MLA, previous data indicated that this unusual expression pattern did not preclude the diagnosis of MLA.

**Figure 6:**
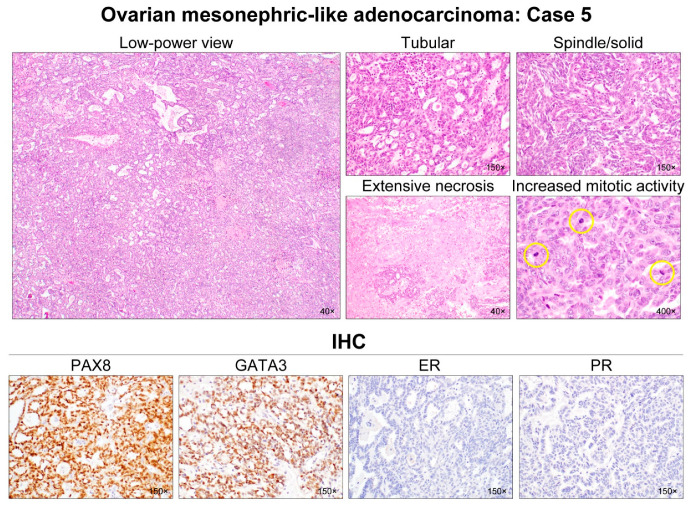
Histological features of ovarian MLA: Case 5. A 6-cm ovarian mass was purely solid and involved the right ovary and lateral uterine wall. A low-power magnification revealed a compact aggregation of numerous small tubules with tiny lumina, resulting in a sieve-like structure. Relatively uniform tubules were lined by columnar epithelial cells and contained dense eosinophilic intraluminal secretions. A few dilated, irregular-shaped ductal structures also possessed pale eosinophilic intraluminal substances. In several areas, the spindle-shaped tumor cells were intermingled with small tubules and showed fascicular and solid growth patterns. They displayed uniform, tapering nuclei. Although most of the tumor cells exhibited mild-to-moderate nuclear pleomorphism without conspicuous nucleoli, some foci showed severe nuclear pleomorphism, extensive coagulative tumor cell necrosis, and increased mitotic activity (yellow circles). This case was diagnosed as stage IIB disease based on the uterine and pelvic peritoneal extension. Immunostaining revealed that the tumor cells were diffusely and strongly positive for PAX8 and GATA3, but completely negative for hormone receptors.

All cases demonstrated relatively uniform, small nuclei with mild-to-moderate pleomorphism. The tumor cells had scant cytoplasm, with low nuclear-to-cytoplasmic ratio. However, two tumors (cases 1 and 5) elicited some foci showing severe nuclear pleomorphism and brisk mitotic activity. Both cases also had scattered areas of extensive coagulative tumor cell necrosis. In addition, one patient (case 1), whose tumors exhibited high-grade nuclear atypia, frequent mitoses, and necrosis, developed distant metastases and peritoneal recurrence. In her metastatic tumor, severe nuclear pleomorphism accompanied by brisk mitotic activity and extensive necrosis was also observed. Representative photomicrographs showing detailed histological features of cases 1–5 are shown in Figure 2, Figure 3, Figure 4, Figure 5 and Figure 6, respectively.

### 3.3. Immunostaining Results

All (5/5) tumors showed diffuse and strong nuclear PAX8 immunoreactivity (Table 4). All cases also exhibited positive GATA3 expression, but their staining intensities and extents were variable: diffuse and strong (2/5); focal and strong (2/5); and focal and moderate (1/5) expression. In one case (case 4), there were abrupt transitions between GATA3-positive and GATA3-negative areas, and both were not morphologically different. PAX2 immunoreactivity was diffuse (4/5) or focal (1/5) with moderate-to-strong intensity. In one tumor (case 4) with focal PAX2 expression, similar to GATA3, there was no significant morphological difference between PAX2-positive and PAX2-negative areas. Four cases showed moderate-to-strong TTF1 expression, whereas all tumors were negative for WT1. Regarding hormone receptors, three tumors displayed patchy nuclear ER expression with weak-to-moderate intensity, while PR expression was identified in only one case with focal positive in some tumor cells. All cases showed wild-type p53 expression pattern and non-diffuse p16 positivity. Of four cases with available tissue for immunostaining of CD10, PTEN, and MMR proteins, CD10 characteristically highlighted the luminal membrane in three cases, and all cases showed no loss of PTEN and MMR protein expression. Representative photomicrographs showing immunostaining results of cases 1–5 are shown in Figure 2, Figure 3, Figure 4, Figure 5 and Figure 6, respectively.

### 3.4. Targeted Sequencing Results

Tumor tissue samples for targeted sequencing were available in four cases (Table 5). All cases showed Activating Kirsten rat sarcoma viral oncogene homolog (*KRAS*) mutations, including c.35G > T (p.G12V; 2/4), c.35G > A (p.G12D; 1/4), and c.34G > T (p.G12C; 1/4). The type of *KRAS* mutation was not associated with specific histological features or immunophenotype. No pathogenic mutation was detected in neuroblastoma rat sarcoma viral oncogene homolog (*NRAS*), tumor protein 53 (*TP53*), Erb-B2 receptor tyrosine kinase 2 (*ERBB2*), AT-rich interaction domain 1A (*ARID1A*), *PTEN*, phosphatidylinositol-4,5-bisphosphate 3-kinase catalytic subunit alpha (*PIK3CA*), and β-catenin (*CTNNB1*).

## 4. Discussion

We investigated the clinicopathological characteristics, immunophenotypes, and molecular alterations of five ovarian MLAs. On imaging studies, one case presented as a purely solid tumor, which was misinterpreted as a mesenchymal tumor of uterine origin. In contrast, four tumors appeared as mixed solid and cystic masses, and were suspected to be primary ovarian borderline tumors or carcinomas. A multilocular cystic mass containing an internal solid component reminds physicians and radiologists of ovarian endometriosis-associated endometrioid carcinoma (EC) or clear cell carcinoma (CCC). Recent studies have documented ovarian MLAs co-existing with Mullerian lesions, such as endometriosis, serous borderline tumor, and low-grade serous carcinoma, supporting the possibility of Mullerian origin for ovarian MLA [7,8,10,11]. Consistent with these findings, we identified either endometriosis or endometriotic cysts in all except one case. Taken together, we found that ovarian MLA can present as either a mixed solid and cystic mass, or a purely solid mass on imaging, and that it can be associated with Mullerian lesions such as endometriotic cysts. Since ovarian MLA does not have any specific imaging finding, pathologists should not exclude the possibility of MLA in the differential diagnosis of ovarian carcinoma until completing a thorough microscopic examination and ancillary tests.

Previous studies revealed 60 cases of ovarian MLA to date, as summarized in Table 5. Half (23/46, 50.0%) of the patients whose stage information were available had stage I tumors, and all 14 patients with available survival data were alive [4,7,10,11,12,14,37]. Pors et al. [9] also reported in their recent multicenter study that the 5-year progression-free survival (PFS) of 23 ovarian MLA patients was 68%. Likewise, our five patients had stage I–II tumors, and three of them had no evidence of recurrent disease. However, accumulating evidence has indicated that most patients with ‘uterine’ MLA initially present with advanced-stage tumors, and develop frequent recurrences and lung metastases [9,15,38]. Pors et al. [9] also reported the 5-year PFS of 43 uterine MLA patients as 27.5%. We also recently demonstrated that 64% (16/25) of uterine MLA patients were diagnosed as having stage III–IV tumors, and 18 (72%) patients experienced post-operative recurrences [39]. Taken together, we prudently assume at least that ovarian MLA might present as a relatively early-stage disease and more favorable prognosis than uterine MLA which is known to exhibit very aggressive clinical behavior and dismal prognosis. Further investigations with larger cohorts are necessary to clarify whether our assumption is reasonable and why ovarian and uterine MLAs demonstrate different outcomes from each other, despite almost identical histomorphologies.

Since there has been no available database to compare the prognosis of MLA with that of other histological types of ovarian carcinoma, it is premature to draw conclusions on the biological behavior of ovarian MLA. With the survival data of the five cases in this study, we focused on the findings indicating the possibility of the aggressive nature of ovarian MLA. In case 1, the patient with stage IA ovarian MLA did not receive any post-operative adjuvant treatment, unlike the other patients. At 13 months post-operatively, she developed abdominopelvic peritoneal carcinomatosis and multiple pulmonary and hepatic metastases (i.e., stage IVB), and subsequently died of disease despite adjuvant treatment. Both the primary and metastatic tumors in this case showed increased mitotic activity (27/10 HPFs) and coagulative tumor cell necrosis. We recently demonstrated that increased mitotic activity (>10/10 HPFs) and tumor necrosis were associated with aggressive biological behavior in uterine MLA [15]. However, it is still insufficient to conclude whether mitotic activity and/or necrosis participate in the recurrence and metastasis of ovarian MLA, because there was another case showing similar histological features but a different clinical presentation. In case 5, similar to case 1, the tumor exhibited easily identifiable mitoses (17/10 HPFs) and extensive necrosis, but the patient is alive without evidence of recurrent or metastatic disease. Although cases 1 and 5 demonstrated early-stage (IA and IIB, respectively) diseases and similar degree of adverse pathological features, recurrence was observed only in case 1 who did not undergo post-operative chemotherapy. Thus, we assumed that post-operative chemotherapy might be necessary for ovarian MLA regardless of the stage. Further investigations are required to reach a consensus for the treatment of ovarian MLAs, especially those with aggressive histology.

Table 6 summarizes the immunostaining results of previously published cases of ovarian MLA, most of which showed nuclear staining for GATA3 (27/29, 93.1%) and TTF1 (26/30, 86.7%). All examined cases exhibited a wild-type p53 expression pattern (19/19), WT1 negativity (0/25), and rare hormonal receptor expression–ER (2/31, 6.5%) and PR (0/25). Our immunostaining results are consistent with the notion that ovarian MLA exhibits expression for mesonephric markers (GATA3, PAX2, TTF1, and CD10) and rare immunoreactivities for hormonal receptors and WT1. A previous study evaluating the usefulness of mesonephric markers in a case series of MA and MLA demonstrated that GATA3 was the best overall marker in terms of high sensitivity (91%) and specificity (94%) [6]. Our results of consistent GATA3 immunostaining in all cases corroborated the previous data. Regarding hormonal receptors, similar to previous studies, our ovarian MLA cases rarely showed ER and PR expression with weak-to-moderate staining intensity [39,40,41,42]. Even though it can be confusing to differentiate MLA from EC with the assumption of the low specificity of ER and PR, low-grade EC mimicking MLA would exhibit well-differentiated tubules or ductal structures showing uniform and strong expression for both ER and PR. When the histological features characteristic of MLA, including compactly aggregated small tubules, eosinophilic dense intraluminal secretions, architectural diversity, and high nuclear-to-cytoplasmic ratio, are observed, it is recommended to perform immunostaining for hormonal receptors and markers of mesonephric lineage to correctly determine the histological type.

Table 7 summarizes the genetic abnormalities of previously published cases of ovarian MLA, and twenty-three of 28 (82.1%) tumors harbored pathogenic *KRAS* mutations, including p.G12D (10/23), p.G12V (10/23), p.G12A (2/23), and p.G12C (1/23). Many recent studies have documented that the *KRAS* mutation is a distinct molecular feature of uterine and ovarian MLA, suggesting that harboring *KRAS* mutation is involved in MLA development [5,10,15,43]. Consistent with previous data, all four tumors whose tumor tissues were available for targeted sequencing harbored pathogenic *KRAS* mutations. Mirkovic et al. [5] performed targeted sequencing using four ovarian and three uterine MLAs, and reported that all examined ovarian MLAs harbored *KRAS* p.G12D, whereas all examined uterine MLAs had *KRAS* p.G12V. However, our targeted sequencing results included p.G12V, p.G12D, and p.G12C, indicating there might be no difference in *KRAS* mutation type according to the primary site of MLA. In the aspect of a clinical implication, frequent *KRAS* mutations could potentially be applied for the treatment of patients with MLA. *KRAS* mutation constitutively results in the activation of the mitogen-activated protein kinase (MAPK) signaling pathway, which subsequently activates downstream substrates, leading to the expression of genes involved in cellular proliferation, differentiation, and survival. As targeted inhibitors of the MAPK pathway have been approved to treat several types of *KRAS*-mutated carcinoma [44], it is worth investigating the therapeutic significance of MAPK inhibitors for patients with MLA. In fact, the effect on growth suppression of MAPK pathway inhibition in ovarian carcinoma cells was more profound in tumors harboring *KRAS* mutation than in *KRAS* wild-type tumors [45].

**Figure 7:**
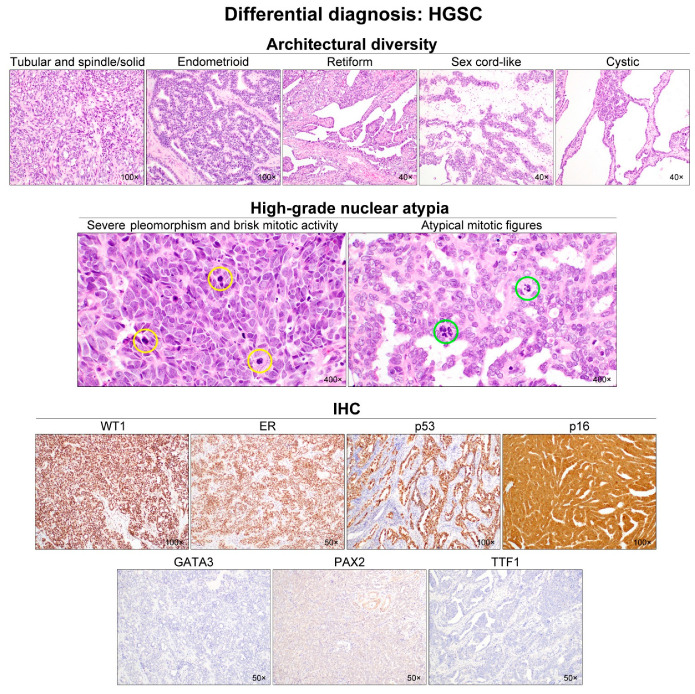
Differential diagnosis of ovarian MLA: HGSC. In addition to papillary and solid structures, HGSC can exhibit various growth patterns including tubular, spindle, endometrioid, retiform, sex cord-like, and cystic. Diverse architectural patterns and the presence of intraluminal secretions may raise the possibility of MLA. However, when a high-grade nuclear atypia, including severe-to-marked nuclear pleomorphism and hyperchromasia, brisk mitotic activity (yellow circles), and readily identifiable atypical mitotic figures (green circles), is observed throughout the tumor, the diagnosis of HGSC is preferred. Nevertheless, as shown in case 1, since MLA can also show severe nuclear pleomorphism and increased mitotic activity, immunostaining is recommended to differentiate between HGSC with mesonephric-like differentiation and MLA. The following immunoprofiles indicate HGSC: uniform nuclear WT1 and ER expression; p53 overexpression; diffuse and strong nuclear and cytoplasmic p16 immunoreactivity; and a lack of mesonephric marker (GATA3, PAX2, and TTF1) expression. MLA has been reported to show negative or patchy p16 expression.

Because of the rarity and diverse histomorphology, an accurate diagnosis of ovarian MLA is often challenging. More common histological types of ovarian carcinoma, including HGSC, EC, and CCC, should be prudently considered in the differential diagnosis as ovarian MLA, since it shares some histological features with those of the tumor subtypes. HGSC can exhibit architectural diversity (Figure 7); however, obvious nuclear atypia throughout the tumor, brisk mitotic activity, diffuse and strong WT1 expression, and mutant p53 immunostaining pattern support the diagnosis of HGSC. Additionally, our recent study demonstrated the pathological and molecular characteristics of HGSC which are distinct from those of MLA [46]: the presence of serous tubal intraepithelial carcinoma (STIC); solid, endometrioid, and transitional (SET) features; solid, endometrioid, transitional, and mucinous-like (STEM) features; and absence of *KRAS* mutation through targeted sequencing. Although STIC is one of the characteristic features of HGSC, there is a case report demonstrating multifocal tubal intraepithelial metastases of ovarian MLA mimicking STIC [37]. Other histological features including obvious nuclear atypia and brisk mitotic activity with appropriate immunostaining results should be preconditional. Likewise, EC is distinguished from MLA by uniform and intense nuclear immunoreactivity for hormonal receptors and the presence of various histological differentiation (squamous, mucinous, and/or sertoliform). Although MLA and CCC both exhibit eosinophilic intraluminal substances, lack of hormonal receptor expression, and wild-type p53 immunostaining pattern, characteristic histological features of CCC (abundant, clear-to-eosinophilic cytoplasm; distinct cell border; enlarged, pleomorphic nuclei with prominent nucleoli; and hyalinized or myxoid stroma) help to distinguish it from MLA [4,47]. CCC typically expresses hepatocyte nuclear factor 1β and napsin A; MLA often exhibits positivity for both markers, so that immunostaining for these proteins would not help to differentiate MLA from CCC. As both MLA and CCC have been reported to harbor co-existing endometriosis, the presence of endometriosis is not useful for the differential diagnosis.

**Figure 8:**
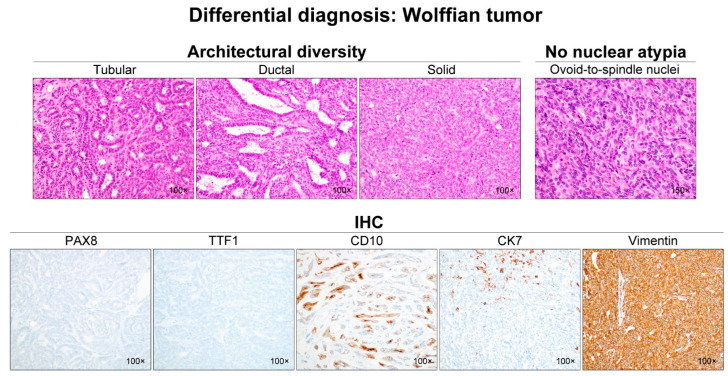
Differential diagnosis of ovarian MLA: Wolffian tumor. Note the variable architectural patterns including tubular, ductal, and solid. The latter pattern is characterized by a spindle cell population. The nuclei are bland, without nucleoli, and have a low mitotic count. The tumor cells of Wolffian tumors are completely negative for PAX8 and TTF1, which are usually expressed strongly by MLA cells. Luminal CD10 staining is similar to that observed in MLA. CK7 shows only focal staining, and vimentin immunoreactivity is diffuse and intense. The expression pattern of CK7 and vimentin in Wolffian tumors is different from that of most ovarian carcinomas, including MLA.

Wolffian tumors (Figure 8), formerly known as female adnexal tumors of probable Wolffian origin, are another rare adnexal tumor with mesonephric differentiation, and should also be included in the differential diagnosis of ovarian MLA. Wolffian tumors typically arise in the leaves of the broad ligament, and are presumed to originate from mesonephric remnants. Similar to MLA, Wolffian tumors exhibit various architectural patterns, including tubular, ductal, cystic, cribriform, sieve-like, sex cord-like, and spindle/solid. The variable-sized tubules and ducts lined by bland-appearing, low columnar-to-cuboidal epithelial cells can possess brightly eosinophilic intraluminal secretions, and the areas showing diffuse or solid growth patterns usually consist of a spindle cell population. However, unlike MLA, it does not react with PAX8, TTF1, GATA3, and epithelial membrane antigen, but expresses vimentin, inhibin, and forkhead box protein L2 [48,49,50]. While MLA shows diffuse and strong membranous cytokeratin 7 (CK7) immunoreactivity, Wolffian tumors are usually focally positive for CK7. Moreover, Wolffian tumors have not been shown to harbor any pathogenic *KRAS* mutation [49,50,51]. As most of adnexal Wolffian tumors have a benign behavior, they should be distinguished from ovarian MLA.

Because of the wide range of morphological diversity, ovarian MLA can possess spindle cells arranged in solid, fascicular, or storiform growth patterns. Moreover, GATA3 has been known to be less reliable when the spindle cell component is present, posing some diagnostic challenges [52,53]. If a considerable portion of MLA tumor tissue demonstrates the spindle cell component, mesonephric-like carcinosarcoma (MLCS) should be included in the differential diagnosis. However, the diagnosis of MLCS should be established when the spindle cells exhibit obvious nuclear atypia, high mitotic index, lack of epithelial marker expression, and gain of vimentin expression [4,6]. The coexistence with heterologous elements also strongly supports the diagnosis of MLCS. In this study, cases 1 and 5 showed a spindle/solid growth pattern, but the nuclear feature of the spindle cell component was similar to that of the adjacent tumor cells with other architectural patterns. There was no obvious nuclear atypia.

## 5. Conclusions

We demonstrated the clinicopathological, immunophenotypical, and genetic features of five ovarian MLAs. All ovarian MLAs were FIGO stage I and II, and all cases with post-operative adjuvant chemotherapy did not develop recurrence. However, one patient with stage IA, without receiving any post-operative adjuvant chemotherapy, developed multiple metastatic recurrences, raising the possibility of aggressive behavior in a subset of ovarian MLA cases and the necessity of post-operative chemotherapy regardless of the initial stage. Ovarian MLA exhibited diverse architectural patterns, compactly aggregated tubules and eosinophilic intraluminal secretions with distinct immunophenotype–positive for mesonephric markers (GATA3, PAX2, TTF1, and CD10), negative WT1 expression, non-diffuse p16 immunoreactivity, and wild-type p53 expression. Genetically, they harbored various types of pathogenic *KRAS* mutations. As ovarian MLA is a rare but clinicopathologically distinct entity, we suggest that pathologists suspect ovarian MLA and make a correct diagnosis with the support of appropriate immunostaining and targeted sequencing.

## Figures and Tables

**Table 1:** Antibodies used.

Antibody	Clone	Company	Dilution
PAX8	Polyclonal	Cell Marque (Rocklin, CA, USA)	1:50
WT1	6F-H2	Cell Marque (Rocklin, CA, USA)	1:800
GATA3	L50-823	Cell Marque (Rocklin, CA, USA)	1:400
TTF1	8G7G3/1	Dako (Agilent Technologies, Santa Clara, CA, USA)	1:100
PAX2	Polyclonal	Invitrogen (Thermo Fisher Scientific, Waltham, MA, USA)	1:100
CD10	56C6	Novocastra (Leica Biosystems, Buffalo Grove, IL, USA)	1:100
ER	6F11	Novocastra (Leica Biosystems, Buffalo Grove, IL, USA)	1:150
PR	16	Novocastra (Leica Biosystems, Buffalo Grove, IL, USA)	1:100
p53	DO-7	Novocastra (Leica Biosystems, Buffalo Grove, IL, USA)	1:200
p16	E6H4	Ventana Medical Systems (Roche, Oro Valley, AZ, USA)	Prediluted
PTEN	SP218	Ventana Medical Systems (Roche, Oro Valley, AZ, USA)	Prediluted
MLH1	M1	Ventana Medical Systems (Roche, Oro Valley, AZ, USA)	Prediluted
PMS2	MRQ-28	Cell Marque (Rocklin, CA, USA)	1:20
MSH2	G219-1129	Cell Marque (Rocklin, CA, USA)	1:500
MSH6	44/MSH6	BD Biosciences (Franklin Lakes, NJ, USA)	1:500

**Table 2:** Clinical features.

Case No.	Age (yrs)	Presenting Symptom	Imaging Finding	CA 125 (U/mL)	CA 19-9 (U/mL)	Clinical Impression	Surgical Treatment	Adjuvant Treatment	Recurrence	Treatment for Recurrence	DFS (mos)	Status	OS (mos)
1	42	Pelvic mass	7.4-cm cystic right ovarian mass with internal solid nodules	72.5	80.1	Ovarian clear cell carcinoma	TH with BSO, PLND, PALND, peritonectomy, and omentectomy (PDS)	–	Lung, liver, and peritoneum	PLND, PALND, peritonectomy, omentectomy, appendectomy, and hepatectomy (SDS), and chemotherapy	13	Dead	39
2	53	–	4.8-cm cystic left ovarian mass with small enhancing nodules	26.8	NA	Ovarian borderline tumor or carcinoma	TH with BSO, PLND, peritonectomy, and omentectomy (PDS)	Chemotherapy	–	–	21	Alive (NED)	21
3	57	Abdominal distension	10.9-cm cystic left ovarian mass with solid component	12.2	25.4	Ovarian borderline tumor or carcinoma	BSO with PLNS, peritonectomy, and omentectomy (PDS)	Chemotherapy	–	–	11	Alive (NED)	11
4	61	Pelvic mass	Solid and cystic left ovarian mass showing intense hypermetabolic activity	NA	NA	Ovarian carcinoma	TH with BSO, PLND, PALND, peritoneal biopsy, and omentectomy (PDS)	Chemotherapy	NA (follow-up loss)	NA	NA	NA	NA
5	52	Pelvic mass	5.8-cm solid mass involving the right lateral uterine wall	108.8	9.3	Uterine leiomyoma or LMS	TH with BSO and peritonectomy (PDS)	Chemotherapy	–	–	53	Alive (NED)	53

Abbreviations: BSO, bilateral salpingo-oophorectomy; CA 19-9, cancer antigen 19-9; CA 125, cancer antigen 125; DFS, disease-free survival; LMS, leiomyosarcoma; mos, months; NA, not applicable; NED, no evidence of disease; OS, overall survival; PALND, para-aortic lymph node dissection; PDS, primary debulking surgery; PLND, pelvic lymph node dissection; PLNS, pelvic lymph node sampling; SDS, secondary debulking surgery; TH, total hysterectomy; yrs, years; –, absent.

**Table 3:** Pathological features.

Case no.	Tumor Location/Size (cm)	Ovarian Surface Extension	Peritoneal Washing Cytology	Uterine Extension	Pelvic Peritoneal Metastasis	Extrapelvic Peritoneal Metastasis	FIGO Stage	Associated Histology	Dominant Growth Pattern	Severe Nuclear Pleomorphism	Mitotic Count (per 10 HPFs)	TCN
1	RO/7.5	–	–	–	– (p); + (r)	– (p); + (r)	IA (p); IVB (r)	–	Ductal, spindle/solid, and tubular	Focal (p); Diffuse (r)	27 (p); 25 (r)	+
2	LO/4.7	+	+	–	–	–	IC	Endometriotic cyst	Tubular, ductal, and sex cord-like	–	6	–
3	LO/11.0	+	–	–	–	–	IC	Endometriotic cyst	Tubular and ductal	–	3	–
4	LO/6.0	–	+	–	–	–	IC	Endometriotic cyst	Ductal, tubular, papillary, and clear	–	5	–
5	RO/6.0	–	NA	+	+	–	IIB	Endometriosis	Tubular andspindle/solid	Focal	17	+

Abbreviations: HPFs, high-power fields; LO, left ovary; NA, not applicable; p, primary; r, recurrent; RO, right ovary; TCN, tumor cell necrosis; +, present; –, absent.

**Table 4:** Immunostaining and targeted sequencing results.

Case No.	PAX8	GATA3 (Nuclear)	PAX2 (Nuclear)	TTF1 (Nuclear)	WT1	ER	PR	p16	p53	CD10 (Luminal)	PTEN	MMR Proteins	KRAS Mutation	Other Mutation
1	+D, S	+F, S	+D, S	+F, S	–	+F, W	–	–	WT	+F, S	No loss	No loss	p.G12V	–
2	+D, S	+F, M	+D, S	+D, S	–	–	–	+P	WT	+F, S	No loss	No loss	p.G12V	–
3	+D, S	+D, S	+D, S	–	–	+F, W	–	+P	WT	+F, M	No loss	No loss	p.G12D	–
4	+D, S	+F, S	+F, S	+F, S	–	+F, M	+F, M	+P	WT	NA	NA	NA	NA	NA
5	+D, S	+D, S	+D, M	+D, M	–	–	–	–	WT	–	No loss	No loss	p.G12C	–

Abbreviations: D, diffuse; F, focal; M, moderate; MMR, mismatch repair; NA, not applicable; P, patchy; S, strong; W, weak; WT, wild-type; +, positive; –, negative.

**Table 5:** Clinical features of previously published cases of ovarian mesonephric-like adenocarcinoma.

Author	No. of Cases	Age (yrs)	Presenting Symptom and Sign	Imaging Finding	Surgical Treatment	FIGO Stage	Recurrence	Status	Survival	OS (mos)
McFarland et al. [4]; Mirkovic et al. [5]	5	42–62 (4/5); NA (1/5)	NA	NA	TH + BSO (2/5); BSO (1/5); NA (2/5)	IA (1/5); IC (1/5); IIB (1/5); IIIC (1/5); NA (1/5)	+(1/5); –(4/5)	PD (1/5); NED (4/5)	Alive (5/5)	7–37 (4/5); NA (1/5)
Pors et al. [6]	1	67	NA	NA	NA	IC	NA	NA	NA	NA
Chapel et al. [7]	1	80	Abdominal pain	12.5-cm pelvic mass, omental cake, mesenteric LNE, multiple hepatic nodules	NAC + TH + BSO + O + P	IIIC	–	NED	Alive	3
McCluggage et al. [8]	5	50–77	Pelvic pain, vaginal discharge (1/5); NA (4/5)	6-cm solid LO mass (1/5); NA (4/5)	TH + BSO + PLND + O + P (1/5); NA (4/5)	IIIA (1/5); NA (4/5)	NA	NA	NA	NA
Pors et al. [9]	25	36–81	Pelvic pain (10/25); uterine bleeding (4/25); abdominal distension or bloating (4/25); incidental (5/25); NA (2/25)	NA	NA	I (11/25); II–IV (7/25); NA (7/25)	+(10/25); –(14/25); NA (1/25)	NA	68% (PFS), 71% (OS) (23/25); NA (2/25)	1–1346
Dundr et al. [10]	1	61	NA	Non-resectable LO mass, hepatic metastases, peritoneal carcinomatosis	NAC + TH + BSO + O + P + A	IVB	–	NED	Alive	NA
Seay et al. [11]	1	67	Pelvic heaviness, polyuria	11-cm complex RA mass	TH + RSO + PLND + O	IA	+	SD	Alive	NA; 18 (RFS)
Chen et al. [12]	1	29	Abdominal discomfort	10-cm solid and cystic RA and 5-cm cystic LA masses	TH + BSO + PLND + PALND + O	IC	–	NED	Alive	13
da Silva et al. [13]	15	36–76	NA	NA	NA	IA (2/15); IC (3/15); IIB (2/15); IIIA (1/15); IIIC (2/15); IV (3/15); NA (2/15)	+(10/15); –(5/15)	NA	NA	NA
Deolet et al. [14]	4	33–75	Acute abdominal pain, hydronephrosis, bilateral pleural effusions (1/4); NA (3/4)	7-cm LO mass (1/4); 15-cm solid and cystic LO mass (1/4); ovarian mass (1/4); 11-cm RO mass (1/4)	LSO (1/4); TH + BSO + O (1/4); BSO (1/4); right partialoophorectomy (1/4)	IA (1/4); IC (1/4); IIIC (1/4); IVB (1/4)	+(1/4); –(3/4)	PR (1/4); NED (3/4)	Alive (4/4)	8–46
Kim et al. [37]	1	47	Pelvic mass	4.4-cm solid and cystic LO mass	PLND + PALND + O+P	IIIC	–	NED	Alive	11

Abbreviations: A, appendectomy; BSO, bilateral salpingo-oophorectomy; LA, left adnexa; LNE, lymph node enlargement; LO, left ovary; LSO, left salpingo-oophorectomy; mos, months; NA, not applicable; NAC, neoadjuvant chemotherapy; NED, no evidence of disease; O, omentectomy; OS, overall survival; P, peritonectomy; PD, progressive disease; PFS, progression-free survival; SD, stable disease; RA, right adnexa; RFS, recurrence-free survival; RO, right ovary; TH, total hysterectomy; yrs, years; +, present; –, absent. Numbers enclosed in parenthesis in each cell indicate the number of positive cases per total cases reported in each article.

**Table 6:** Immunostaining results of previously published cases of ovarian mesonephric-like adenocarcinoma.

Author	No. of Cases	GATA3 (Nuclear)	WT1	ER	PR	p16	p53	PTEN	CD10 (Luminal)	TTF1 (Nuclear)
McFarland et al. [4]; Mirkovic et al. [5]	5	+D (1/5); +F (1/5); –(2/5); NA (1/5)	–(5/5)	–(5/5)	–(5/5)	+F (2/5); NA (3/5)	WT (3/5); NA (2/5)	NA (5/5)	+F (2/5); –(1/5); NA (2/5)	+D (4/5); +F (1/5)
Pors et al. [6]	1	+D, M-S	NA	–	NA	NA	NA	NA	+FS	+FM-S
Chapel et al. [7]	1	+	–	–	–	+F	WT	NA	+F	+
McCluggage et al. [8]	5	+F (1/5); NA (4/5)	–(1/5); NA (4/5)	–(1/5); NA (4/5)	–(1/5); NA (4/5)	NA	WT (1/5); NA (4/5)	NA	+D (1/5); NA (4/5)	+F (1/5); NA (4/5)
Pors et al. [9]	25	NA	NA	NA	NA	NA	NA	NA	NA	NA
Dundr et al. [10]	1	+D	–	–	–	NA	WT	NA	+F	+D
Seay et al. [11]	1	+F	–	–	–	+F	WT	No loss	+F	+F
Chen et al. [12]	1	+	NA	–	–	–	WT	NA	+	+
da Silva et al. [13]	15	+(14/15); NA (1/15)	–(12/15); NA (3/15)	+F (2/15); –(13/15)	–(11/15); NA (4/5)	NA	WT (7/15); NA (8/15)	NA	+(2/15); –(3/15); NA (10/15)	+(11/15); –(4/15)
Deolet et al. [14]	4	+D (3/4); +F (1/4)	–(3/4); NA (1/4)	–(4/4)	–(3/4); NA (1/4)	NA	WT (3/4); NA (1/4)	NA	+D (2/4); –(1/4); NA (1/4)	+D (1/4); +F (3/4)
Kim et al. [37]	1	+D	–	–	–	+P	WT	No loss	NA	NA
Total [4,5,6,7,8,9,10,11,12,13,14,37]	60	+(27/29); –(2/29)	–(25/25)	–(29/31); +F (2/31)	– (25/25)	+F (5/6); –(1/6)	WT (19/19)	No loss (2/2)	+(12/17); –(5/17)	+(26/30); –(4/30)

Abbreviations: D, diffuse; F, focal; M, moderate; NA, not applicable; P, patchy; S, strong; WT, wild-type; +, positive; –, negative. Numbers enclosed in parenthesis in each cell indicate the number of positive cases per total cases reported in each article.

**Table 7:** Genetic features of previously published cases of ovarian mesonephric-like adenocarcinoma.

Author	No. of Cases	*KRAS* Mutation	Other Recurrent Mutation	Recurrent Chromosomal Gain	Recurrent Chromosomal Loss
McFarland et al. [4]; Mirkovic et al. [5]	5	p.G12D (4/5); NA (1/5)	*PIK3CA*	1q	1p
Pors et al. [6]	1	NA	NA	NA	NA
Chapel et al. [7]	1	–	*NRAS*, *BCOR*	1q, 4, 7, 13, 18p, 21	1p, 11, 18q, 19, 22, X
McCluggage et al. [8]	5	p.G12D (1/5); NA (4/5)	–	NA	NA
Pors et al. [9]	25	NA	NA	NA	NA
Dundr et al. [10]	1	p.G12C	*PIK3CA*, *CHEK2*	–	–
Seay et al. [11]	1	–	–	–	–
Chen et al. [12]	1	NA	NA	NA	NA
da Silva et al. [13]	15	p.G12D (5/15); p.G12V (8/15); –(2/15)	*PIK3CA*, *SPOP*, *NRAS*, *SETD8*, *CTNNB1*, *CREBBP*, *NOTCH3*, *ARID1A*, *FBXW7*, *FANCA*, *AKT1*, *ASXL1*, *RAD54L*	1q, 2, 6, 7, 8, 9, 10, 12, 16, 17, 20, 21, X	1p, 4, 6, 15, 18, 22, X
Deolet et al. [14]	4	p.G12A (2/4); p.G12V (1/4); –(1/4)	*PIK3CA*, *PTEN* amplification, 12p isochromosome	1q, 12, 17, 21	–
Kim et al. [37]	1	p.G12V	–	–	–
Total [4,5,6,7,8,9,10,11,12,13,14,37]	60	p.G12D (10/28); p.G12V (10/28); p.G12A (2/28); p.G12C (1/28); –(5/28)			

Abbreviations: –, absent. Numbers enclosed in parenthesis in each cell indicate the number of positive cases per total cases reported in each article.

## Data Availability

Not applicable.
